# Sex differences in imaging features including cerebral amyloid angiopathy markers in intracerebral hemorrhage

**DOI:** 10.3389/fstro.2026.1810711

**Published:** 2026-06-29

**Authors:** Trine Apostolaki-Hansson, Christine Kremer, Amir Hillal, Cheryl Carcel, Teresa Ullberg, Bo Norrving, Jesper Petersson

**Affiliations:** 1 Department of Neurology, Skåne University Hospital, Malmö/Lund, Sweden; 2 Department of Clinical Sciences Lund, Lund University, Lund, Sweden; 3 Department of Neuroradiology, Karolinska University Hospital, Stockholm, Sweden; 4 The George Institute for Global Health, University of New South Wales, Sydney, NSW, Australia; 5 Prince of Wales Clinical School, University of New South Wales, Sydney, NSW, Australia

**Keywords:** ICH, intracerebral hemorrhage, neuroradiology, sex differences, stroke, subarachnoid extension

## Abstract

**Background:**

Reports on sex differences in radiological characteristics of intracerebral hemorrhage (ICH) are few. Sex-related differences in hematoma location, volume, and imaging markers may contribute to variations in clinical presentation and outcome. We aimed to assess sex differences in non-contrast computed tomography (NCCT) features in an unselected ICH cohort.

**Methods:**

This observational study included 1,398 patients with spontaneous supratentorial ICH from the Skåne Hospital Region, Sweden (2016–2021), registered in Riksstroke. Radiological characteristics were compared between males and females. Multivariable logistic regression, adjusted for confounders, analyzed sex differences overall and stratified by hematoma location (deep/lobar). CAA probability was assessed using the simplified Edinburgh CT criteria.

**Results:**

Among 785 males and 613 females, hematoma volume, location, and antithrombotic use were similar. Women were older (79 vs. 73 years; *p* < 0.001), more often had severe white matter changes on baseline NCCT, with overall differences in white matter change distribution between sexes (*p* = 0.006), intraventricular hemorrhage (45.2% vs. 38.7%; *p* = 0.02), finger-like projections (18.1% vs. 10.6%; *p* < 0.001), subarachnoid extension (25.1% vs. 15.7%; *p* < 0.001), and hydrocephalus (16.6% vs. 10.0%; *p* = 0.001). In lobar ICH (*n* = 666), high CAA probability was more common in women (28.8% vs. 15.0%, *p* < 0.001), and in multivariable analysis, female sex was independently associated with subarachnoid extension (OR 1.89 95%CI 1.29–2.77).

**Conclusion:**

In this large, unselected cohort of supratentorial ICH, no sex differences were observed in hematoma volume, location, or intraventricular extension. However, in lobar ICH, female sex was independently associated with subarachnoid extension and CT features suggestive of higher CAA probability. These findings indicate similar hemorrhage severity between sexes but differences in lobar hemorrhage morphology that require further validation and explanation.

## Introduction

Reports on sex differences in the radiological characteristics of intracerebral hemorrhage (ICH) remain limited. Although sex-related differences are well-established in ischemic stroke (Carcel et al., 2019; Kelly-Hayes et al., 2003; Caso et al., 2010), considerably less is known in hemorrhagic stroke (Rexrode et al., 2022), particularly ICH. Biological factors, including hormonal differences, may influence hemorrhage distribution, morphology, and expansion on neuroimaging. However, existing evidence regarding radiological features of ICH is inconsistent (Roquer et al., 2016; Gokhale et al., 2015).

While ICH incidence is lower in females than in males (Foschi et al., 2024), findings on hemorrhage characteristics are conflicting. A previous study suggested similar etiology, severity, and hemorrhage volume between the sexes, although females had a higher frequency of lobar ICH (Roquer et al., 2016). In contrast, two North American cohorts observed a male predominance of deep ICH but no sex differences in lobar location (Labovitz et al., 2005; Flaherty et al., 2005). An individual patient data meta-analysis (*n* = 4,812) further found that male sex was associated with non-lobar location, larger hematoma volumes, and a higher risk of hematoma expansion (Rivier et al., 2025).

Findings regarding hematoma volume remain mixed. One study found no sex differences in baseline hematoma volume (14.9 vs. 13.6 ml, *p* = 0.053; Witsch et al., 2024), whereas another reported smaller volumes in females (9.9 vs. 11.3 ml, *p* = 0.004; Sandset et al., 2020). Similarly, a Japanese cohort observed no sex difference in hematoma location (Inagawa et al., 2003).

Moreover, sex differences in the incidence of cerebral amyloid angiopathy (CAA) are not fully understood and studies using MRI imaging according to the Boston Criteria version 2.0 have not identified any difference in the prevalence of CAA between sexes (Koemans et al., 2024; Incontri et al., 2025). A recent study determined no difference in clinical and imaging characteristics between sexes including cerebral small vessel disease as well as probable/possible cerebral amyloid angiopathy (CAA) according to Boston criteria v2.0 (Incontri et al., 2025).

We aimed to characterize sex differences in non-contrast computed tomography (NCCT) features of spontaneous supratentorial ICH, in a large, unselected cohort. Furthermore, the distribution of probable CAA according to the simplified Edinburgh CT criteria was explored in a subgroup of lobar ICH patients.

## Methods

### Study population and database

This observational cohort study included patients aged ≥18 years with spontaneous non-traumatic intracerebral hemorrhage (ICD-10 I61), registered in the Swedish Stroke Register (Riksstroke) between January 1, 2016–December 31, 2020. Data were collected from eight primary stroke centers and one comprehensive stroke center in the Skåne region. Patients were included if a baseline NCCT image was available in the regional Picture Archiving and Communication System (PACS). Patients with ICH caused by trauma, an underlying brain tumor, or arterial aneurysms were not included in this study. In Riksstroke, patients with aneurysmal subarachnoid hemorrhage (SAH) or isolated non-aneurysmal, including convexity SAH are not classified as ICH and are not included in this study cohort. Riksstroke is a hospital-based quality register for stroke care in Sweden, providing nationwide coverage of >90% of all hospitalized stroke cases. To determine mortality status and dates of death, data from the Swedish Cause of Death Register, a registry with nearly 100% completeness, were used.

### Study outcome variables

Patients were categorized based on biological sex, female or male, and demographic as well as baseline clinical and radiological characteristics were documented during the acute stroke period. Pre-stroke independence was defined as individuals who managed dressing, toileting, and indoor mobility without assistance and did not rely on homecare services. Pre-stroke dependency included individuals who required homecare support, lived in assisted living facilities, or needed assistance with daily activities such as dressing, toileting, and mobility.

Stroke severity at admission was assessed by patient level of consciousness (LOC) at hospital admission using the reaction level scale (RLS-85), a widely applied tool in Sweden that aligns closely with the Glasgow Coma Scale (Starmark et al., 1988). Patients were categorized into three LOC groups: alert (RLS 1), drowsy (RLS 2–3), and comatose (RLS 4–8). Additional clinical variables collected included the use of oral anticoagulant (OAC) reversal treatment and the occurrence of any neurosurgical intervention, both of which were recorded from 2017 onwards. Data on 90-day functional outcome, assessed using the modified Rankin Scale (mRS), and mortality were obtained from Riksstroke.

Neuroimaging data were derived from NCCT scans, retrieved via the regional PACS, with radiological data from the nine hospitals in the Skåne region included in this study, using a validated ICH database (Sultani et al., 2024). Imaging was performed on scanners from all major manufacturers, with thin axial reconstructions (0.5–1 mm slice thickness) used for analysis. Hematoma volumes included the parenchymal component of the ICH, and any intraventricular extension and/or subarachnoid extension. Volumes were quantified using manual segmentation with the Sectra Volume Measurement tool (Sectra IDS7, Sectra, Linköping, Sweden) and cross-validated using the ABC/2 method. NCCT scans for patients with supratentorial ICH were initially evaluated by a radiology resident with 1 year of neuroradiology experience. To ensure reliability, the first 500 scans, including 375 supratentorial ICH cases, underwent independent review by a senior neuroradiologist with >20 years of experience for inter-rater agreement.

Image evaluation included the assessment of ICH location (lobar, deep, and/or intraventricular), lateralization, single vs. multifocal hemorrhage (defined as multiple ICHs with no connection), the presence of SAH extension, finger-like projections (FLP), presence of known or newly diagnosed vascular malformation at the time of stroke onset, subdural components, intraventricular extension, midline shift (millimeters), hydrocephalus, and qualitative grading of white matter changes on NCCT was performed as follows: mild (punctate hypodensities in the periventricular or deep white matter without confluence), moderate (partial confluence of hypodense areas), and severe (extensive, confluent hypodensities extending into deep or subcortical white matter).

### Statistical methods

Statistical analyses were conducted using IBM SPSS Statistics version 27. Baseline characteristics are presented as proportions for categorical variables and medians with interquartile ranges for continuous variables. Proportions were derived from frequency tables, and group differences between males and females were assessed using the chi-squared test for categorical variables and the Mann–Whitney *U* test for continuous variables. Cohen's kappa was used to assess inter-rater agreement for hematoma volume as well as the presence of SAH and FLP on NCCT images.

We performed logistic regression analyses to determine associations between sex and radiological imaging findings. Initial univariable models were used to assess the relationship between sex and individual imaging parameters. Variables that showed statistical significance in univariable analyses and/or were identified priorly as clinically relevant were included in the multivariable logistic regression models, adjusting for potential confounders. Three separate logistic regression models were constructed for patients with deep, lobar, and all spontaneous supratentorial ICH to assess associations between sex and radiological imaging features. In the primary analysis, female sex was modeled as the dependent variable (female = 1, male = 0) to identify imaging features associated with sex. The models were adjusted for age, hypertension, diabetes mellitus, previous stroke, oral anticoagulant treatment, antiplatelet treatment, statin use, hematoma volume, subarachnoid extension, intraventricular extension, and degree of white matter changes on NCCT. Furthermore, in the logistic regression model including all spontaneous supratentorial ICH cases, lobar vs. deep hematoma location was also added as a covariate in the regression analysis. OAC reversal treatment was not included in multivariable models, as data were only available from 2017 onwards, timing of anticoagulation and reversal was unknown, and the variable is subject to substantial confounding by indication as indicated in our previous study (Apostolaki-Hansson et al., 2020). Odds ratios (OR) with corresponding 95% confidence intervals (CI) were reported for all models. A two-sided *p*-value < 0.05 was considered statistically significant.

Cerebral amyloid angiopathy probability was assessed in patients with lobar intracerebral hemorrhage using the simplified Edinburgh CT criteria (Sembill et al., 2022). Patients were classified into three categories: low probability (lobar ICH without associated SAH), intermediate probability (lobar ICH with associated SAH), and high probability (lobar ICH with associated SAH and the presence of FLP). The distribution of CAA probability categories was compared between men and women using the chi-squared test.

To explore whether the relationship between sex and hematoma volume varied by age, we conducted a subgroup analysis stratified by age groups ( ≤ 64, 65–74, 75–84, and ≥85 years). Logistic regression was performed within each age stratum to evaluate potential age–sex interactions. In exploratory, *post-hoc* analyses, logistic regression models were performed in the total cohort, a female-only, and in lobar-only population subgroups to assess whether SAH extension was associated with poor outcome at 90 days, defined as a modified Rankin Scale (mRS) score of 3–6.

This study adheres to the Strengthening the Reporting of Observational Studies in Epidemiology (STROBE) checklist (von et al., 2007).

### Ethical considerations

The approval of this study was granted by the Swedish Ethical Review Authority (dnr 2020-04680).

## Results

A total of 1,398 patients with spontaneous supratentorial ICH were included (785 males and 613 females). Baseline demographic, clinical, and imaging features are presented in Table 1. Inter-rater reliability was excellent for hematoma location (lobar vs. deep; κ = 0.84) and moderate for SAH extension in lobar ICH (κ = 0.55) and FLP (κ = 0.49).

**Table 1:** Baseline characteristics of 1,398 patients with spontaneous supratentorial intracerebral hemorrhage.

Variables	Male (*n* = 785)	Female (*n* = 613)	*P*-value
Demographics			
Age (median IQR)	73 (65–81)	79 (71–85)	<0.001
Living alone prior to ICH	274 (36.0)	341 (57.3)	<0.001
Prestroke dependent	120 (16.8)	162 (30.1)	<0.001
Vascular risk factors			
Diabetes mellitus	159 (20.3)	106 (17.3)	0.15
Hypertension	457 (58.6)	371 (61.5)	0.27
Atrial Fibrillation	210 (27.0)	144 (23.7)	0.16
Prior stroke	203 (25.9)	145 (23.8)	0.38
Lipid-lowering agent	258 (33.1)	163 (27.1)	0.02
Antiplatelet drug at onset	212 (27.2)	149 (24.7)	0.29
Oral anticoagulant drug at onset	203 (25.9)	145 (23.7)	0.34
DOAC			
*Apixaban*	47/203 (23.2)	45/145 (31.0)	0.10
*Dabigatran*	12/203 (5.9)	7/145 (4.8)	0.66
*Rivaroxaban*	37/203 (18.2)	22/145 (15.2)	0.45
*VKA*	110/203 (54.2)	71/145 (49.0)	0.34
OAC reversal treatment (2017 onwards)	117/164 (71.3)	74/121 (61.2)	0.07
Stroke and clinical characteristics			
Level of consciousness			0.02
*Alert*	465 (59.7)	317 (52.3)	
*Drowsy*	188 (24.1)	166 (27.4)	
*Comatose*	126 (16.2)	123 (20.3)	
Stroke unit admission	663 (84.5)	515 (84.0)	0.82
ICU admission	110 (14.0)	67 (10.9)	0.09
Length of hospital stay (median days–IQR)	10 (5–21)	10 (4–17)	0.01
Neurosurgical intervention (2017 onwards)	46 (7.3)	23 (4.6)	0.06
Symptom onset to hospital arrival			0.77
≤ 3 h	43 (15.6)	37 (15.3)	
≤ 6 h	127 (46.0)	122 (50.4)	
≤ 24 h	89 (32.2)	69 (28.5)	
>24 h	17 (6.2)	14 (5.8)	
Imaging characteristics			
CT angiography	158 (20.1)	91 (14.8)	0.01
MRI brain performed during time of care	102 (13.0)	72 (11.8)	0.49
Hematoma volume (median–IQR)	17 (4–46)	21 (5–56)	0.09
Hematoma volume			0.44
< 10 ml	286 (36.4)	211 (34.3)	
10–29 ml	202 (25.7)	148 (24.1)	
30–79 ml	190 (24.2)	153 (25.0)	
≥80 ml	107 (13.6)	101 (16.5)	
Hemorrhage location			0.13
*Lobar*	360 (45.9)	306 (49.9)	
*Deep*	425 (54.1)	307 (50.1)	
Intraventricular hemorrhage extension	304 (38.7)	277 (45.2)	0.02
Subarachnoid extension	123 (15.7)	154 (25.1)	<0.001
Finger-like projections	83 (10.6)	111 (18.1)	<0.001
Hydrocephalus			0.001
*Beginning*	111 (14.2)	91 (14.9)	
*Manifest*	78 (10.0)	101 (16.6)	
Midline shift millimeters (median–IQR)	0 (0–5)	2 (0–7)	0.003
White matter changes			0.006
*None*	182 (23.2)	107 (17.5)	
*Mild*	287 (36.6)	206 (33.6)	
*Moderate*	89 (11.3)	81 (13.2)	
*Severe*	227 (28.9)	219 (35.7)	
Vascular malformation^**^	14 (1.8)	8 (1.3)	0.48
Follow-up CT during hospital stay	300 (38.2)	168 (27.4)	<0.001
Death within 90 days	242 (30.8)	219 (35.7)	0.05

Missing data were < 2% for all variables, except for prestroke dependency (10.3%) and symptom onset to hospital arrival (63.0%).

ICH, intracerebral hemorrhage; ICU, intensive care unit; IQR, interquartile range; DOAC, direct oral anticoagulant drug; OAC, oral anticoagulant; VKA, vitamin K antagonist.

^**^does not include aneurysms.

Hematoma volume and location distribution did not differ between sexes. However, females more frequently had IVH extension (45.2% vs. 38.7%; *p* = 0.02), SAH extension (25.1% vs. 15.7%; *p* < 0.001), and hydrocephalus (16.6% vs. 10.0%; *p* = 0.001). The overall distribution of white matter changes on NCCT also differed between females and males (*p* = 0.006), with severe white matter changes being more common in females (35.7% vs. 28.9%). Hemorrhage extension into the subarachnoid space was primarily seen in patients with lobar ICH (lobar ICH *n* = 666/1,398). Among these, 42.8% of females (131/306) had SAH extension compared to 28.6% of males (103/360; *p* < 0.001). In lobar ICH, finger-like projections were also more common in females compared to males (18.1% vs. 10.6%; *p* < 0.001).

In lobar ICH (*n* = 666), multivariable logistic regression showed higher odds of SAH extension in females (OR 1.71 95%CI 1.24–2.36), whereas no significant sex differences were observed in deep ICH (*n* = 732; Tables 2, 3). Hematoma location (lobar vs. deep) was not associated with sex in the overall cohort (Supplementary Table S1). In exploratory analysis using logistic regression, SAH extension was not associated with poor functional outcome (mRS 3–6) in the overall cohort (OR 1.63 95%CI 0.75–3.55; *n* = 1,398), in a female-only cohort (OR 1.09 95%CI 0.33–3.54; *n* = 613), or in a lobar-only cohort (OR 2.05 95%CI 0.91–4.62; *n* = 666; Supplementary Tables S2–S4).

**Table 2:** Multivariable logistic regression analysis of radiological imaging features associated with female sex in lobar intracerebral hemorrhage (*n* = 666)^*^.

Variable	OR	95% CI		*P*-value
		Lower	Upper	
Subarachnoid extension	1.89	1.29	2.77	0.001
IVH extension	1.14	0.73	1.79	0.57
Hematoma volume (*ref < 10 ml*)				
*10–29 ml*	0.66	0.42	1.05	0.08
*30–79 ml*	0.78	0.48	1.27	0.32
*≥80 ml*	0.64	0.34	1.21	0.17
NCCT verified white matter changes *(none = ref)*				
*Mild*	0.69	0.43	1.10	0.12
*Moderate*	0.89	0.46	1.71	0.73
*Severe*	0.56	0.33	0.94	0.03

CI, confidence interval; ICH, intracerebral haemorrhage; IVH, intraventricular haemorrhage; NCCT, non-contrast computed tomography; OR, odds ratio. Ref, reference.

^*^Female sex was modeled as the dependent variable (female = 1, male = 0). Model adjusted for age, hypertension, diabetes mellitus, previous stroke, oral anticoagulant treatment, antiplatelet therapy, and statin use.

**Table 3:** Multivariable logistic regression analysis of radiological imaging features associated with female sex in deep intracerebral hemorrhage (*n* = 732)^*^.

Variable	OR	95% CI		*P*-value
		Lower	Upper	
Subarachnoid extension	1.44	0.72	2.90	0.30
IVH extension	1.27	0.87	1.86	0.22
Hematoma volume (*ref < 10 ml*)				
*10–29 ml*	1.15	0.76	1.73	0.51
*30–79 ml*	0.93	0.57	1.50	0.76
*≥80 ml*	0.98	0.52	1.83	0.94
NCCT verified white matter changes (*none = ref*)				
*Mild*	0.99	0.59	1.67	0.98
*Moderate*	1.08	0.57	2.05	0.81
*Severe*	1.50	0.84	2.69	0.17

CI, confidence interval; ICH, intracerebral haemorrhage; IVH, intraventricular hemorrhage; NCCT, non-contrast computed tomography; OR, odds ratio. Ref, reference.

^*^Female sex was modeled as the dependent variable (female = 1, male = 0). Model adjusted for age, hypertension, diabetes mellitus, previous stroke, oral anticoagulant treatment, antiplatelet therapy, and statin use.

Table 4 shows CAA probability in lobar intracerebral hemorrhage (*n* = 666) according to the simplified Edinburgh CT criteria. Low probability was more common in men than women (71.4% vs. 57.2%), intermediate probability was similar (13.6% vs. 14.1%), and high probability was more frequent in women (28.8% vs. 15.0%). Overall distributions differed significantly by sex (*p* < 0.001).

**Table 4:** Cerebral amyloid angiopathy probability in lobar intracerebral hemorrhage according to the simplified Edinburgh CT criteria (*n* = 666).

Classification	Male (*n* = 360)	Female (*n* = 306)	*p*-value
	*n* (%)	*n* (%)	
**Low CAA probability**	257 (71.4)	175 (57.2)	<0.001
*Lobar ICH, without associated SAH*			
**Intermediate CAA probability**	49 (13.6)	43 (14.1)	
*Lobar ICH with associated SAH*			
**High CAA probability**	54 (15.0)	88 (28.8)	
*Lobar ICH with associated SAH and presence of FLP*			

CAA, cerebral amyloid angiopathy; CT, computed tomography; FLP, finger like projections; ICH, intracerebral hemorrhage; SAH, subarachnoid hemorrhage.

**Figure 1:**
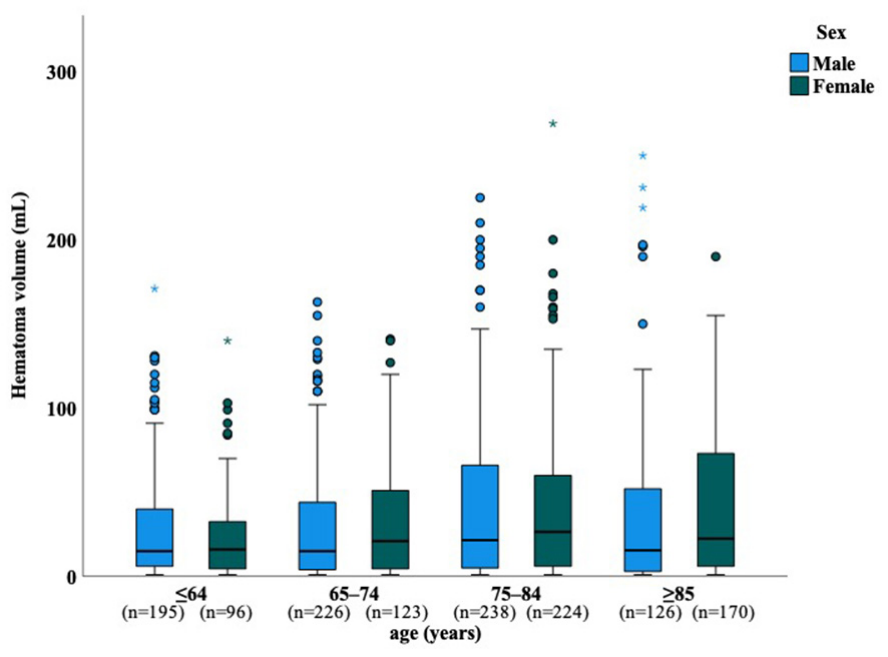
Distribution of hematoma volume (mL) by sex across age groups (≤64, 65–74, 75–84, and ≥85 years) in patients with intracerebral hemorrhage. Box plots display the median (central line), interquartile range (box), and whiskers extending to the most extreme values within 1.5× the interquartile range. Comparisons between sexes within each age group were performed using the Mann–Whitney *U* test. Corresponding *p*-values were: ≤64 (*p* = 0.52), 65–74 (*p* = 0.37), 75–84 (*p* = 0.71), and ≥85 years (*p* = 0.11).

In age-stratified analyses (Figure 1), females ≤ 64 years were less likely than males to present with very large hematomas (≥80 ml; OR 0.28 95%CI 0.08–0.96; *p* = 0.04; Supplementary Table S5).

## Discussion

In this large, unselected observational cohort study of patients with spontaneous supratentorial ICH, our primary aim was to investigate whether sex differences exist in radiological presentation on baseline NCCT. Overall, we observed no meaningful sex differences in hematoma volume, lobar vs. deep location, or IVH extension in the total population. In subgroup multivariable analysis restricted to patients with lobar ICH (*n* = 666), female sex was independently associated with SAH extension on initial NCCT. These findings suggest that men and women present with similar radiological features in terms of classical markers for hemorrhage severity and anatomical localization, but that certain morphological features of lobar hemorrhage may differ.

Presence of subarachnoid extension in lobar ICH most commonly represents extension of a superficially located hematoma into the cortical subarachnoid space, supplied by fragile leptomeningeal arterioles, and is strongly associated with CAA (Charidimou et al., 2015). The higher frequency of SAH extension seen in females in our cohort therefore supports the possibility of sex-related differences in the underlying vasculature. Several biological mechanisms may contribute. Estrogen supports endothelial stability, preserves blood-brain barrier integrity, and suppresses matrix metalloproteinases that degrade vascular walls (Robison et al., 2019), and its decline after menopause may increase small vessel fragility. Females also have higher cerebral blood flow throughout life (Rodriguez et al., 1988) and age-related microvascular structural differences, including greater microvessel density (Hohmann et al., 2024), which may predispose to rupture of superficial cortical vessels. Structural differences between sexes as those described above may be a contributing factor to the higher rate of SAH extension found in female lobar ICH patients in the present cohort (42.8% vs. 28.6%; *p* < 0.001).

Sex differences in the incidence of CAA are not fully understood, and studies have not identified any difference in the prevalence of CAA between sexes (Koemans et al., 2024; Incontri et al., 2025). Since SAH extension and FLPs are a part of the simplified Edinburgh CT criteria as imaging markers associated with CAA, we further explored CT features consistent with higher CAA probability. In our cohort, women with lobar ICH more frequently demonstrated CT features consistent with a high probability of CAA according to the simplified Edinburgh CT criteria. Our results may suggest that women with lobar ICH are more likely to have CAA-related hemorrhage rather than sex-related differences in radiological presentation alone. This interpretation is supported by a recent validation study showing that cortical involvement, SAH extension, and FLPs, when assessed together using the simplified Edinburgh CT criteria, correlate strongly with CAA confirmed by MRI according to the Boston Criteria (Grangeon et al., 2023). These findings are important as CAA-related hemorrhages have a high risk of recurrence and influence decisions regarding secondary prevention, including reinstatement of antithrombotic therapy. However, as SAH extension and FLPs are components of the simplified Edinburgh criteria, analyses of SAH and CAA probability are not independent. Consequently, the observed association between female sex and higher CAA probability is most likely driven by these same imaging features identified in the primary analyses, rather than representing an independent finding.

Women ≤ 64 years were less likely than men to present with very large hematomas (≥80 mL), suggesting that factors influencing hemorrhage volume may differ at younger ages, while morphological differences such as SAH extension appear more prominent in older patients. This finding suggests that both biological differences and vascular changes with aging may contribute to differing hemorrhage patterns between sexes.

In an earlier study, we determined that female sex was associated with poorer 3-month functional status (mRS 3–5), and that male sex was associated with a higher case-fatality following spontaneous supratentorial ICH (Apostolaki-Hansson et al., 2025). The presence of SAH is of importance because it may be associated with poorer neurological outcomes (Wolfert et al., 2022). However, exploratory analysis did not show a significant association between SAH extension and poor outcome at 90 days (mRS 3–6). The point estimates for SAH extension in female- and lobar-only populations suggest a possible association with poor outcome [female-only (*n* = 613) OR 2.25 95%CI 0.66–7.67; lobar-only (*n* = 666) OR 2.05 95%CI 0.91–4.62], but the wide CIs indicate uncertainty, probably due to limited statistical power. Moreover, as lobar ICH is generally associated with lower mortality compared to deep hemorrhages (Hillal et al., 2025), the presence of SAH extension within these subgroups may not independently be associated with worse outcome.

This study has several strengths, including the large, unselected cohort with radiological assessment enhancing the generalisability of the study. Limitations include the retrospective design with potential residual confounding. Since women were substantially older than men, and several imaging features of interest (e.g., WMH, SAH extension, and CAA probability) are strongly age-related. Consequently, residual confounding cannot be excluded despite multivariable adjustment. Secondly, the absence of MRI-based markers of CAA and small vessel disease which could better characterize underlying CAA pathology. Third, CAA probability was assessed using CT-based criteria rather than MRI, the gold standard. Consequently, analyses of SAH and CAA probability are not fully independent, and caution is warranted when interpreting associations between sex, SAH extension, and CAA probability. Fourth, inter-rater agreement for SAH and FLP was moderate. However, similar moderate agreements were seen in previous studies of the simplified Edinburgh CT criteria (Sembill et al., 2022). Misclassification of SAH and FLP may have influenced the observed associations with sex, however, the direction of this potential bias is uncertain. Fifth, a further limitation is that female sex was modeled as the dependent variable. While this approach identifies imaging features associated with sex, it does not support causal interpretation, and the direction of association should be interpreted with caution. Another limitation of the study is that the registry variable for neurosurgical treatment included all types of neurosurgical interventions and did not allow differentiation between hematoma evacuation and other procedures. In addition, neurosurgical intervention rates were low in the cohort, which may have influenced overall prognosis. However, the frequency of neurosurgical intervention was similar between males and females. Lastly, subgroup analysis regarding hematoma volume stratified by age categories and sex should be interpreted with caution as the sample size per category was small.

## Conclusion

In this large, unselected cohort of spontaneous supratentorial ICH, we found no sex differences in hematoma volume, location, or intraventricular extension on baseline NCCT. However, among patients with lobar ICH, female sex was independently associated with SAH extension and CT imaging markers suggestive of higher CAA probability. These findings suggest that sex may influence hemorrhage morphology rather than hemorrhage severity and support heightened evaluation for underlying CAA in women with lobar ICH. Further research is required to validate our findings.

## Data Availability

The datasets presented in this article are not readily available because “Data is available on request and after approval from Riksstroke.” Requests to access the datasets should be directed to trine.apostolaki-hansson@med.lu.se.
