# Supplementary material for: Sex differences in imaging features including cerebral amyloid angiopathy markers in intracerebral hemorrhage
Source: Front Stroke. 2026 Jun 29;5:1810711. doi: 10.3389/fstro.2026.1810711 (PMC13356935; doi:10.3389/fstro.2026.1810711)
Supplement: Supplementary file 1 [file Table_1.docx]

| Supplemental Table 1. Multivariable logistic regression analysis of radiological imaging features associated with female sex in spontaneous supratentorial intracerebral hemorrhage (n = 1398) * | | | | |
| --- | --- | --- | --- | --- |
| Variable | OR | 95% CI | | P-value |
|  |  | lower | upper |  |
| Hematoma volume *(ref <10ml)* |  |  |  |  |
| *10-29 ml* | 0.92 | 0.68 | 1.24 | 0.57 |
| *30-79 ml* | 0.91 | 0.65 | 1.27 | 0.56 |
| *≥80 ml* | 0.79 | 0.51 | 1.23 | 0.29 |
| Hematoma location *(ref lobar)* |  |  |  |  |
| *Deep* | 1.01 | 0.78 | 1.30 | 0.96 |
| Subarachnoid extension | 1.71 | 1.24 | 2.36 | 0.001 |
| IVH extension | 1.26 | 0.95 | 1.68 | 0.11 |
| NCCT verified white matter changes *(none=ref)* |  |  |  |  |
| *mild* | 0.78 | 0.55 | 1.10 | 0.16 |
| *moderate* | 0.92 | 0.59 | 1.43 | 0.70 |
| *severe* | 0.87 | 0.59 | 1.27 | 0.47 |
| Abbreviations: CI=confidence interval, ICH=intracerebral hemorrhage, IVH=intraventricular hemorrhage, NCCT=non-contrast computed tomography, OR=odds ratio. Ref=reference.  *Female sex was modelled as the dependent variable (female=1, male=0). Model adjusted for age, hypertension, diabetes mellitus, previous stroke, oral anticoagulant treatment, antiplatelet therapy, and statin use. | | | | |

| Supplemental Table 2. Multivariable logistic regression analysis of 90-day poor functional outcome, defined as a modified Rankin Scale score of 3–6, following spontaneous intracerebral hemorrhage in the total cohort (n = 1398). | | | | |
| --- | --- | --- | --- | --- |
| Variable | OR | 95% CI | | P-value |
|  |  | lower | upper |  |
| Age | 1.07 | 1.04 | 1.09 | <0.001 |
| Female sex | 0.88 | 0.54 | 1.43 | 0.60 |
| Prior stroke | 2.66 | 1.34 | 5.29 | 0.005 |
| Hypertension | 1.12 | 0.66 | 1.91 | 0.68 |
| Diabetes mellitus | 1.53 | 0.83 | 2.82 | 0.18 |
| Oral anticoagulant therapy | 0.60 | 0.31 | 1.15 | 0.12 |
| Antiplatelet therapy | 0.60 | 0.31 | 1.19 | 0.15 |
| Statin use | 0.89 | 0.48 | 1.63 | 0.70 |
| Hematoma volume *(ref <10ml)* |  |  |  |  |
| *10-29 ml* | 2.22 | 1.29 | 3.81 | 0.004 |
| *30-79 ml* | 10.30 | 4.81 | 22.06 | <0.001 |
| *≥80 ml* | 30.42 | 6.28 | 147.42 | <0.001 |
| Subarachnoid extension | 1.63 | 0.75 | 3.55 | 0.22 |
| IVH extension | 1.99 | 1.09 | 3.63 | 0.03 |
| Deep location (lobar reference) | 3.51 | 2.07 | 5.95 | <0.001 |
| Neurosurgical intervention | 1.20 | 0.45 | 3.22 | 0.72 |
| NCCT verified white matter changes *(none=ref)* |  |  |  |  |
| *mild* | 1.17 | 0.65 | 2.13 | 0.60 |
| *moderate* | 0.86 | 0.37 | 2.02 | 0.73 |
| *severe* | 1.97 | 0.91 | 4.27 | 0.08 |
| Abbreviations: CI=confidence interval, IVH=intraventricular hemorrhage, NCCT=non-contrast computed tomography, OR=odds ratio. Ref=reference. | | | | |

| Supplemental Table 3. Multivariable logistic regression analysis of 90-day poor functional outcome, defined as a modified Rankin Scale score of 3–6, following spontaneous intracerebral hemorrhage in a female-only population (n = 613). | | | | |
| --- | --- | --- | --- | --- |
| Variable | OR | 95% CI | | P-value |
|  |  | lower | upper |  |
| Age | 1.07 | 1.02 | 1.12 | 0.003 |
| Prior stroke | 2.90 | 0.63 | 13.42 | 0.17 |
| Hypertension | 0.98 | 0.39 | 2.42 | 0.96 |
| Diabetes mellitus | 1.79 | 0.55 | 5.84 | 0.33 |
| Oral anticoagulant therapy | 0.38 | 0.12 | 1.26 | 0.11 |
| Antiplatelet therapy | 0.43 | 0.12 | 1.62 | 0.21 |
| Statin use | 1.70 | 0.52 | 5.51 | 0.38 |
| Hematoma volume *(ref <10ml)* |  |  |  |  |
| *10-29 ml* | 1.84 | 0.70 | 4.83 | 0.21 |
| *30-79 ml* | 14.62 | 3.12 | 68.55 | 0.001 |
| *≥80 ml* | 20.17 | 1.91 | 212.91 | <0.001 |
| Subarachnoid extension | 1.09 | 0.33 | 3.54 | 0.89 |
| IVH extension | 3.17 | 1.00 | 10.04 | 0.05 |
| Deep location (lobar reference) | 4.03 | 1.58 | 10.31 | 0.004 |
| Neurosurgical intervention | 0.58 | 0.10 | 3.19 | 0.53 |
| NCCT verified white matter changes *(none=ref)* |  |  |  |  |
| *mild* | 2.01 | 0.73 | 5.59 | 0.18 |
| *moderate* | 1.10 | 0.26 | 4.74 | 0.90 |
| *severe* | 4.41 | 1.17 | 16.65 | 0.03 |
| Abbreviations: CI=confidence interval, ICH=intracerebral hemorrhage, IVH=intraventricular hemorrhage, NCCT=non-contrast computed tomography, OR=odds ratio. Ref=reference. | | | | |

| Supplemental Table 3. Multivariable logistic regression analysis of 90-day poor functional outcome, defined as a modified Rankin Scale score of 3–6, following spontaneous intracerebral hemorrhage in a lobar-only population (n = 666). | | | | |
| --- | --- | --- | --- | --- |
| Variable | OR | 95% CI | | P-value |
|  |  | lower | upper |  |
| Age | 1.04 | 1.003 | 1.08 | 0.03 |
| Female sex | 0.70 | 3.56 | 1.38 | 0.30 |
| Prior stroke | 2.47 | 1.00 | 6.06 | 0.05 |
| Hypertension | 0.90 | 0.42 | 1.94 | 0.79 |
| Diabetes mellitus | 1.67 | 0.63 | 4.38 | 0.30 |
| Oral anticoagulant therapy | 0.75 | 0.31 | 1.84 | 0.53 |
| Antiplatelet therapy | 0.69 | 0.29 | 1.61 | 0.39 |
| Statin use | 0.82 | 0.37 | 1.84 | 0.63 |
| Hematoma volume *(ref <10ml)* |  |  |  |  |
| *10-29 ml* | 1.34 | 0.62 | 2.90 | 0.46 |
| *30-79 ml* | 6.25 | 2.50 | 15.63 | <0.001 |
| *≥80 ml* | 21.27 | 3.85 | 117.41 | <0.001 |
| Subarachnoid extension | 2.05 | 0.91 | 4.62 | 0.08 |
| IVH extension | 1.07 | 0.42 | 2.74 | 0.89 |
| Neurosurgical intervention | 0.60 | 0.19 | 1.88 | 0.38 |
| NCCT verified white matter changes *(none=ref)* |  |  |  |  |
| *mild* | 1.28 | 0.58 | 2.86 | 0.54 |
| *moderate* | 0.57 | 0.17 | 1.86 | 0.35 |
| *severe* | 2.40 | 0.89 | 6.47 | 0.08 |
| Abbreviations: CI=confidence interval, ICH=intracerebral hemorrhage, IVH=intraventricular hemorrhage, NCCT=non-contrast computed tomography, OR=odds ratio. Ref=reference. | | | | |

| Supplemental Table 5. Logistic regression analysis showing Odds Ratios for the effect of female sex on hematoma volume following intracerebral hemorrhage on initial non-contrast computed tomography, stratified by age (n = 1398). | | | | |
| --- | --- | --- | --- | --- |
| Variable | OR | 95% CI | | P-value |
|  |  | lower | upper |  |
| **Age ≤64** **(n=291)** |  |  |  |  |
| *ref <10 ml (n=106)* | 1 | - | - | - |
| *10-29 ml (n=89)* | 1.04 | 0.54 | 2.00 | 0.92 |
| *30-79 ml (n=71)* | 0.54 | 0.25 | 1.20 | 0.13 |
| *≥80 ml (n=25)* | 0.28 | 0.08 | 0.96 | 0.04 |
| **Age 65–74 (n=349)** |  |  |  |  |
| *ref <10 ml (n=134)* | 1 | - | - | - |
| *10-29 ml (n=83)* | 1.01 | 0.54 | 1.88 | 0.98 |
| *30-79 ml (n=87)* | 0.78 | 0.40 | 1.54 | 0.47 |
| *≥80 ml (n=45)* | 0.62 | 0.23 | 1.63 | 0.33 |
| **Age 75–84 (n=462)** |  |  |  |  |
| *ref <10 ml (n=154)* | 1 | - | - | - |
| *10-29 ml (n=103)* | 0.67 | 0.38 | 1.17 | 0.16 |
| *30-79 ml (n=124)* | 0.98 | 0.55 | 1.76 | 0.94 |
| *≥80 ml (n=81)* | 0.87 | 0.42 | 1.80 | 0.70 |
| **Age ≥85 (n=296)** |  |  |  |  |
| *ref <10 ml (n=103)* | 1 | - | - | - |
| *10-29 ml (n=75)* | 1.14 | 0.60 | 2.16 | 0.69 |
| *30-79 ml (n=61)* | 1.33 | 0.61 | 2.88 | 0.47 |
| *≥80 ml (n=57)* | 1.49 | 0.59 | 3.74 | 0.40 |
| Adjusted for age, hypertension, diabetes mellitus, previous stroke, oral anticoagulant treatment, antiplatelet treatment, lobar vs. deep hematoma location, subarachnoid extension, intraventricular extension, and white matter changes.  Abbreviations: ICH = intracerebral hemorrhage, OR = odds ratio, CI = confidence interval. Ref = reference. | | | | |
